# Comparison of the anti-inflammatory effects of Cilomilast, Budesonide and a p38 Mitogen activated protein kinase inhibitor in COPD lung tissue macrophages

**DOI:** 10.1186/2050-6511-13-15

**Published:** 2012-11-13

**Authors:** Marianne Jennifer Ratcliffe, Iain Gordon Dougall

**Affiliations:** 1 Personalised Healthcare and Biomarkers, AstraZeneca R&D Alderley Park, Cheshire, SK, 10 4TG, UK; 2 IGD Consultancy Limited, Loughborough, LE, 11 3JR, UK

**Keywords:** COPD, Lung, Macrophage, TNF, Budesonide, Steroid insensitivity, p38 MAPK, PDEIV, BIRB-796

## Abstract

Chronic Obstructive Pulmonary Disease (COPD) is a disease characterized by a largely irreversible airflow obstruction and a persistent, excessive inflammatory response. Alveolar macrophages (AMs) are increased in the lungs of COPD patients, and act as orchestrators of the inflammatory response, releasing a range of mediators to coordinate recruitment and activation of leukocytes. Attempts to treat the inflammatory component of COPD with anti-inflammatory drugs such as steroids has met with limited success. In this study, we compared the ability of the phosphodiesterase IV (PDEIV) inhibitor Cilomilast, the steroid Budesonide, and the p38 mitogen activated protein kinase inhibitor BIRB-796 to inhibit tumour necrosis factor alpha (TNFα) and interleukin 6 (IL-6) releases from AMs isolated from COPD lung transplant tissue. All studies were carried out with appropriate ethical approval and written, informed consent was obtained from each subject. Cilomilast had little effect on cytokine release from AMs. There was considerable variability in the responsiveness of AMs to Budesonide, with a subset of AMs responding poorly to Budesonide. BIRB-796 inhibited TNFα release from all AM donors, including those that responded poorly to steroids. Treatment with BIRB-796 and Budesonide together gave an additive decrease in TNFa release. These results suggest that a p38 inhibitor may provide advantages over existing anti-inflammatory treatments for COPD, either as an add-on to existing therapy, or to treat patients who respond poorly to steroids.

## Background

COPD is an increasingly prevalent disease, affecting up to 10% of adults aged over 40 years [1]. Current therapies include long acting β_2_-receptor agonists (LABAs) and muscarinic receptor antagonists, which increase lung function by relaxing airway smooth muscle. Corticosteroids are also used, and have been shown to decrease exacerbations as well as improving other clinical parameters such as FEV_1_[2]. However, the use of inhaled steroids in COPD is somewhat controversial due to inconsistent clinical effects and reports that these agents have limited effects on lung inflammation in COPD patients. For example, a meta-analysis of the effects of inhaled corticosteroids on inflammatory cells in the sputum of stable COPD patients showed evidence of reductions in neutrophils and lymphocytes but no effect on macrophages [3]. A similar analysis conducted recently on bronchial biopsies and bronchoalveolar lavage (BAL) fluid from stable patients showed reductions in neutrophils and lymphocytes in BAL but an increase in macrophages. The biopsy analysis indicated no effect on neutrophil and macrophage counts but a reduction in CD4^+^ and CD8^+^ lymphocytes [4]. In accordance with these results, macrophages from COPD patients have been reported to be insensitive to steroids [5,6]. Various mechanisms for this steroid insensitivity have been proposed, including up-regulation of NF-kB signaling and increased oxidative stress [7]. If specific mechanisms are indeed responsible for the poor efficacy of steroids in COPD, then alternative anti-inflammatory approaches may be more successful. One such approach currently being considered is inhibition of PDEIV, by drugs such as Roflumilast and Cilomilast. The former drug has recently been approved as an add-on therapy for the maintenance treatment of severe COPD in the European Union and as a treatment to reduce the risk of exacerbations in the United States. These cAMP elevating agents have been shown to reduce recruitment of macrophages and CD8^+^ T-cells in COPD biopsies [8] and improve FEV_1_, alone or in combination with a bronchodilator therapy [9,10]. p38 MAP kinase is involved in transducing a number of inflammatory stimuli [11] and inhibitors of this enzyme have broad anti-inflammatory potential. Indeed, p38 inhibitors have shown evidence of anti-inflammatory effects and improvements in clinical parameters in COPD patients [12,13]. Such agents have also been shown to inhibit cytokine release from human AMs derived from patients with COPD [14,15], but the effectiveness of p38 inhibitors, steroids and PDEIV inhibitors has not been directly compared in the same donors.

In the present study we compared the ability of Cilomilast, Budesonide and BIRB-796 to inhibit cytokine release from AMs isolated from patients undergoing COPD lung transplant surgery for severe end stage disease (GOLD IV). This allowed us to directly compare the effectiveness of different mechanisms in modulating cytokine release, and assess donor to donor variability in response.

## Results

### Characterisation of LPS response

**Figure 1:**
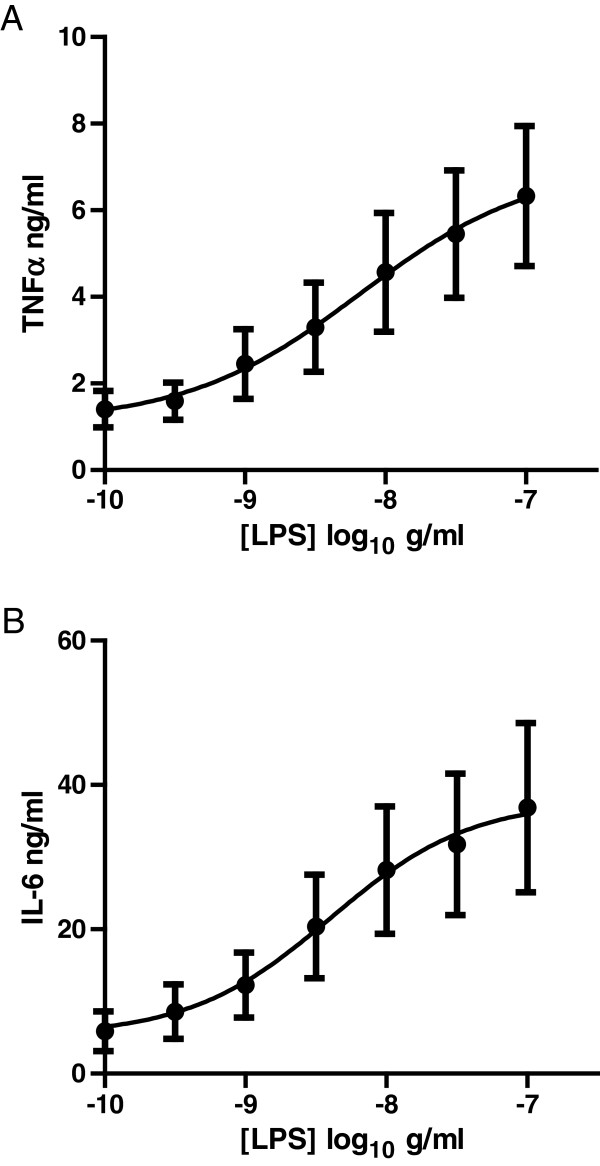
**TNFα (A) and IL-6 (B) release in response to LPS stimulation in COPD lung macrophages as measured by ELISA (n =11).** Data is expressed as mean ± s.e.

A concentration-effect curve to LPS was generated in AMs from eleven COPD transplant donors, measuring both IL-6 and TNFα release. All donors responded to LPS, with potency values of p[A]_50_ = 8.2 (6 ng/ml) for TNFα and 8.3 (4 ng/ml) for IL-6. Meaned maximal response was 6.3 ± 1.6 ng/ml for TNFα and 34.1 ± 7.2 ng/ml for IL-6 (Figure 1). These cytokine levels were similar or greater to that seen in COPD macrophages used in similar published studies [14,15]. The concentration of LPS (100 ng/ml) subsequently used for testing the effects of the anti-inflammatory agents corresponded to a maximum response for both cytokine readouts. 

### Response of COPD AMs to Cilomilast

**Figure 2:**
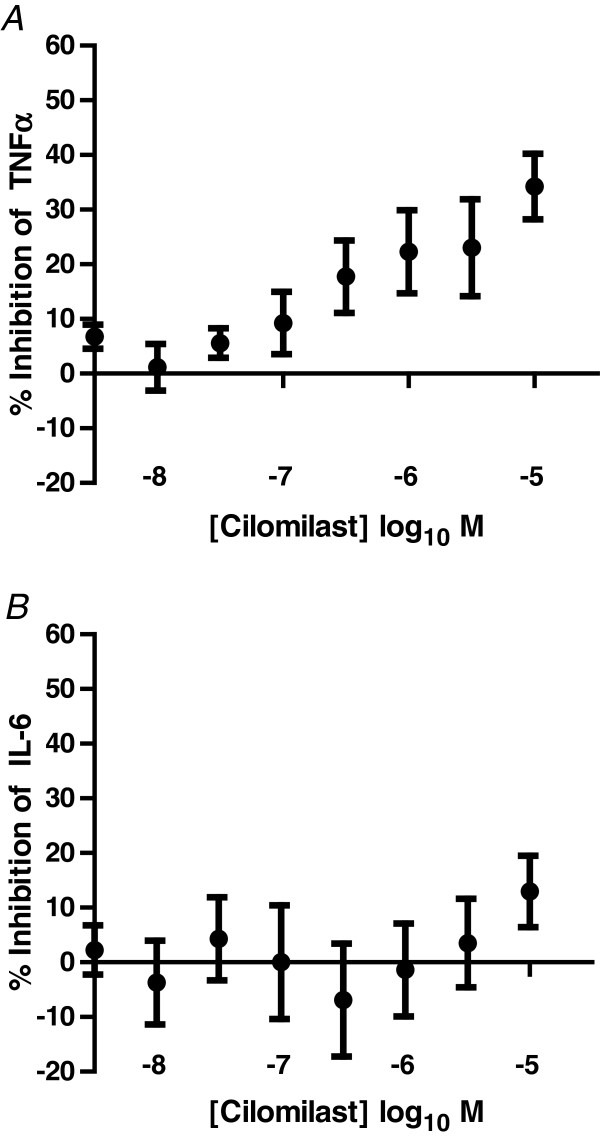
**Effect of Cilomilast on LPS-induced TNFα (A) or IL-6 (B) release from COPD lung macrophages (n =11).** Data is expressed as mean ± s.e.

The ability of Cilomilast, a PDEIV inhibitor, to inhibit LPS-induced cytokine release was tested. Cilomilast did not inhibit IL-6 release at any of the concentrations used (Figure 2B), and only inhibited TNFα release at concentrations greater than 300 nM (Figure 2A) reaching maximum levels of inhibition of 34.2 ± 6.0 at 10 μM.

### Anti-inflammatory effects of Budesonide in COPD AMs

**Figure 3:**
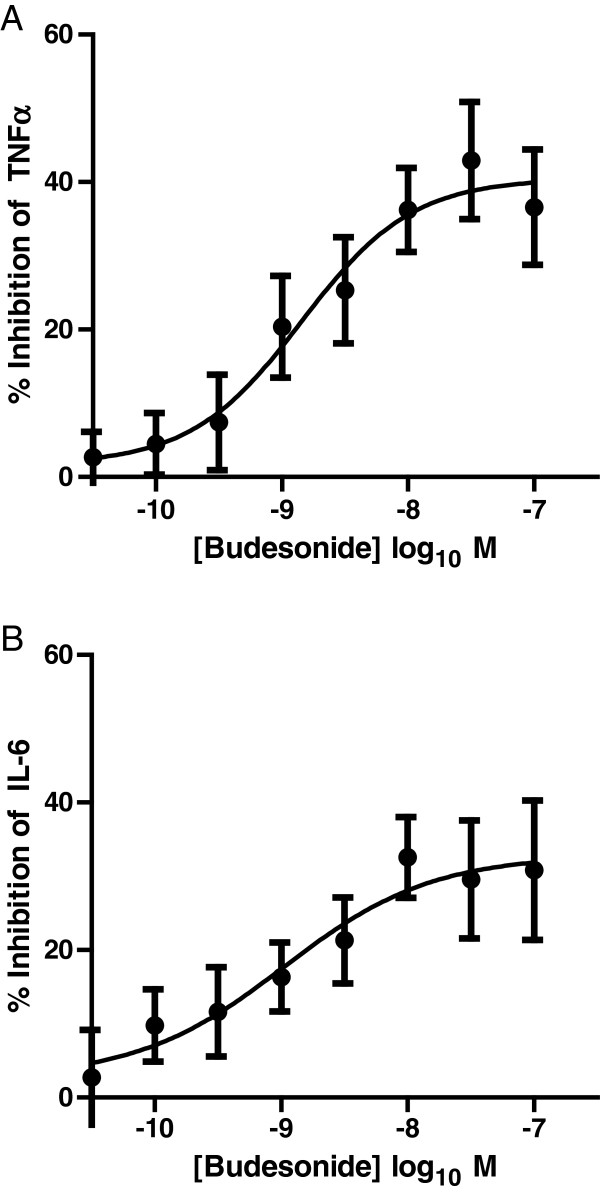
**Effect of Budesonide on LPS-induced TNFα (A) or IL-6 (B) release from COPD lung macrophages (n = 11).** Data is expressed as mean ± s.e. and the lines drawn through the points are the result of fitting using equation 2.

The ability of the steroid Budesonide to inhibit TNFα or IL-6 release from COPD AMs was tested. Budesonide inhibited TNFα release by a maximum of 42.9% ± 8.0%, with a pIC_50_ of 8.9 (1.3 nM). A similar potency was seen against IL-6 (pIC_50_ = 9.0), although the maximum effect was lower than for TNFα (30.8% ± 9.4%) (Figure 3).

### Response of COPD AMs to BIRB-796

**Figure 4:**
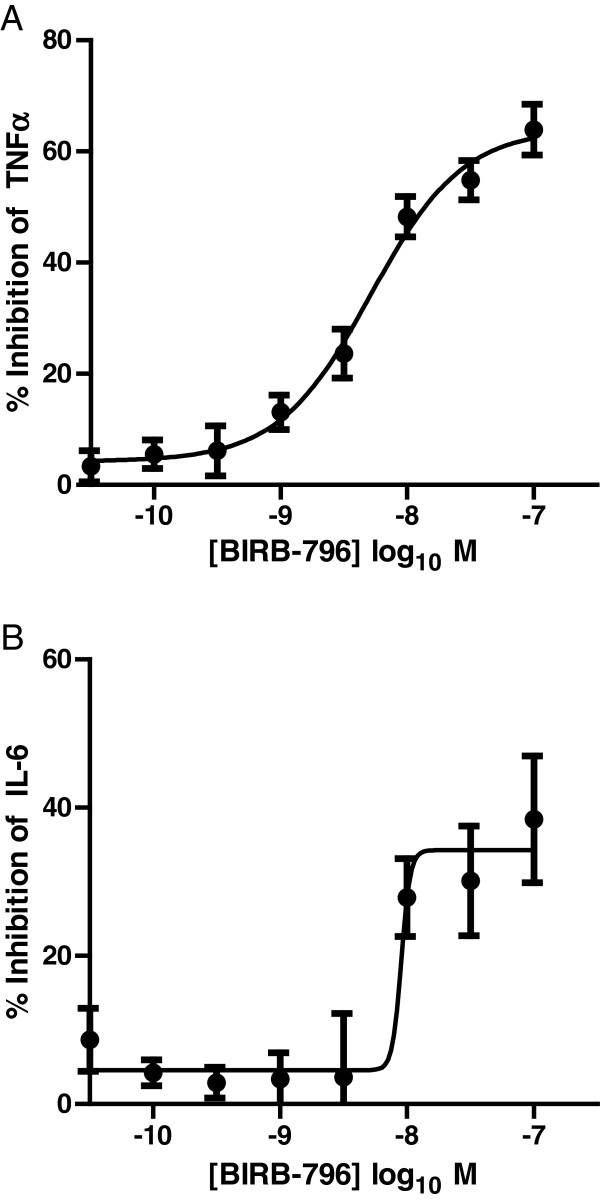
**Effect of BIRB-796 on LPS-induced TNFα (A) or IL-6 (B) release from COPD (lung macrophages (n = 11).** Data is expressed as mean ± s.e. and the lines drawn through the points are the result of fitting using equation 2.

The ability of BIRB-796, a p38 inhibitor, to inhibit macrophage cytokine release was also tested. BIRB-796 inhibited TNFα with a pIC_50_ = 8.3 (5 nM) and a maximum inhibition of 63.9% ± 4.6% (Figure 4A). Effects on IL-6 release were less marked and more variable, resulting in a relatively poor fit to equation 2. The maximum inhibition achieved was 38.4% ± 8.6%, and a pIC_50_ estimated as 8.2 (6.3 nM).

### BIRB-796 responses in steroid-resistant donors

**Figure 5:**
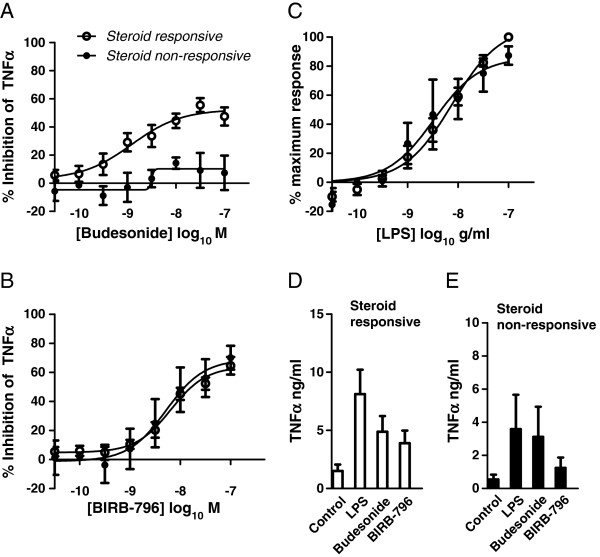
**The effects of Budesonide (A) and BIRB-796 (B) in COPD AMs separated into donors resistant (< 30% inhibition of LPS-induced TNFα release, n = 3), closed circles) or sensitive (> 30% inhibition, n = 8, open circles) to steroid.** (**C**) LPS concentration-effection response curve in steroid resistant (closed circles) or steroid sensitive (open circles) COPD donors. Data is expressed as mean ± s.e. and the lines drawn through the points are the result of fitting using equations 1 (LPS data) and 2 (Budesonide and BIRB-796 data). Absolute cytokine release levels for steroid responsive (**D**) and steroid non-responsive (**E**) groups in response to LPS alone and in the presence of 100 nM Budesonide or BIRB-796.

Although the AMs from the majority of COPD donors responded to Budesonide, we observed considerable variability in the potency and maximum effect of Budesonide between different donors. For inhibition of TNFα, the average standard deviation across the concentration range in COPD donors was 21.3% for Budesonide. In comparison, the p38 response was much less variable, with an average standard deviation of 12.4%. Of note, three of the COPD donors were either unresponsive, or poorly responsive to steroid as defined by showing less than 30% inhibition of LPS-induced TNFα release at the maximal concentration of Budesonide used. The data for these three donors were separated from the remaining donors and re-plotted (Figure 5A). The inhibitory effect of BIRB-796 in these three steroid refractory cells was similar to the steroid responsive AMs (Figure 5B). Thus the ability of BIRB-796 to inhibit TNFα release was maintained in cells with poor steroid sensitivity. We confirmed that the potency of the LPS response in the steroid resistant donors was not significantly different from that of the steroid sensitive donors, ruling out the possibility that shift in the potency of LPS could have explained the differential response to steroid (Figure 5C). The average levels of TNFα release from the steroid responsive donors appeared higher than in the non-responsive, but since the groups are small the significance of this is unclear. The range of TNFα release in the steroid non-responsive group (0.6-7.6 ng/ml) lay within the range seen in the steroid responsive group (0.3-15.4 ng/ml).

### Combination of BIRB-796 and budesonide

**Figure 6:**
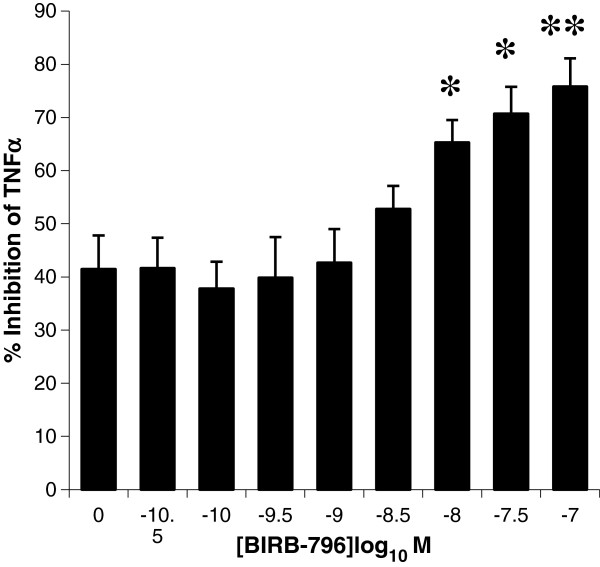
**Combined effect of 100 nM Budesonide in the presence of increasing concentration of BIRB-796 on LPS induced TNFa release from COPD lung macrophages (n=5).** A two tailed T-test was performed to assess statistical significance. * *p* values <0.05, ** p value <0.01.

Combination therapy is an increasing trend within the respiratory area, in an attempt to increase the therapeutic efficacy of medications. The combined effect of Budesonide and BIRB-796 was assessed in COPD AMs. Using a maximally effective concentration of Budesonide (100 nM), an additive effect of BIRB-796 on the release of TNF in response to LPS was observed (Figure 6).

## Discussion

We have investigated the pharmacological profile of three different anti-inflammatory agents in COPD lung macrophages. We used LPS as a stimulus, given the strong links between bacterial colonization and exacerbations of COPD [16]. TNF and IL-6 are both pleiotropic, pro-inflammatory cytokines which are elevated in COPD patients [17,18]. Furthermore, genetic polymorphisms in both these cytokines have been linked to development of COPD [19,20]. The response to the PDEIV inhibitor, Cilomilast, was poor, consistent with published data showing limited effects of PDEIV inhibitors in inhibiting cytokine production from human macrophages [21,22]. Such data suggests that suppression of macrophage function is not a key contributor to the observed clinical efficacy of PDEIV inhibitors in COPD, which may instead lie with anti-inflammatory effects on other cells such as neutrophils or epithelial cells. Alternatively, the modest potency of cilomilast may have limited the effects of this agent and therefore it would be interesting to evaluate the properties of other PDEIV inhibitors. The steroid Budesonide and the p38 inhibitor BIRB-796 were effective anti-inflammatory agents in alveolar macrophages although their effectiveness was dependent on the particular cytokine readout. TNFα release was significantly inhibited by both compounds, but IL-6 was more resistant to inhibition. Other studies have also demonstrated efficacy of steroids in reducing cytokine release from COPD macrophages, with the magnitude of the effect varying between readouts [15,23] . In our study, AMs exhibited a broad spectrum of sensitivities to Budesonide ranging from one donor which failed to show any inhibition of cytokine release, to donors in which the steroid gave over 75% inhibition of TNFα release. This data suggests that cellular steroid insensitivity may not be characteristic of COPD. Rather, there appears to be a significant proportion of individuals whose show a poor cellular response to steroid. Increasingly, physicians and payers are looking towards personalized healthcare approaches, so that individuals likely to respond or fail to respond to treatment can be identified. Steroid treatment is linked to a range of serious side effects, and if those patients who are steroid insensitive could be identified, an alternative treatment option could be selected, thus avoiding unnecessary exposure to steroid.

Of particular interest is our observation that BIRB-796 inhibited TNFα release from AMs equally well in COPD donors that were good or poor responders to Budesonide. This data indicates that p38 inhibitors might be effective in patients which respond poorly to steroids. p38 MAPK pathways have been shown to be active in COPD [24] and a p38 inhibitor has been shown to down-regulate a different panel of mediators to steroids, which may also provide an advantage in a disease setting [15]. Although a number of oral p38 MAPK inhibitors have ceased development due to unwanted side-effects, inhaled p38 inhibitors may have an acceptable therapeutic window and thus represent useful new anti-inflammatory agents. Indeed, PF-03715455 is being developed as an inhaled agent for the treatment of COPD [25]. Such agents could be considered as steroid replacements, or as a second-line treatment option in patients with a poor response to steroid. Recent studies have demonstrated additive effects of steroids and p38 inhibitors in reducing cytokine release from bronchoalveolar lavage (BAL) macrophages and PBMCs from asthmatics [26] and COPD patients [27]. Our data confirms and extends these results, demonstrating additive effects of BIRB-796 and Budesonide in macrophages from a different compartment (lung tissue versus BAL) and to severe (GOLD stage IV) COPD patients, as compared to mild/moderate disease. Thus, our data adds to a growing body of evidence suggesting that a combination of steroid plus p38 inhibitor, on the background of standard bronchodilator therapy, could deliver increased clinical efficacy in severe COPD patients.

## Conclusions

In a subset of subjects with GOLD IV stage COPD, steroids are ineffective in reducing cytokine release from tissue macrophages, yet the inhibitory response to the p38 MAPK inhibitor BIRB-796 is maintained in these cells. Use of inhaled p38 MAPK inhibitors may therefore provide a more effective therapy than steroids in some COPD patients. In addition, combination of steroid with a p38 inhibitor provides additive anti-inflammatory effects in COPD lung tissue macrophages.

## Methods

### Reagents

LPS (*E. coli* 026:B6), Budesonide and Foetal Calf Serum (FCS) were from Sigma-Aldrich, Poole, Dorset UK. Cilomilast and BIRB-796 was synthesised by the Medicinal Chemistry Department, AstraZeneca R&D Charnwood. RPMI, DMEM, Iscoves modified Dulbecco’s medium containing GlutaMAX™ (IMDM), L-glutamine and Penicillin/Streptomycin were from Invitrogen Ltd, Paisley UK. Compounds were made up in Dimethyl Sulfoxide (DMSO) at 1000x final concentration, such that the final concentration of DMSO was 0.1%.

### Subjects

All studies were approved by the Northumberland Local Research Ethics Committee (REC reference 06/Q0902/57). Written, informed consent was obtained from each subject. The demographic and lung function data for the COPD transplant patients are summarised in Table 1. All patients had severe end stage disease and a diagnosis of emphysema.

**Table 1:** Subject information. Data is average ± s.e

	**Sex (M/F)**	**Age (years)**	**FEV**_**1**_**(l)**	**FEV**_**1**_**/FVC (%)**
COPD transplant patients (n = 11)	6/5	52.9 ± 2.4	0.6 ± 0.1	28.3 ± 6.9

### Cytokine release assays

Human AMs were obtained from COPD patients undergoing lung transplant. Macrophages were flushed from the tissue with phosphate buffered saline (PBS), then plated at 100,000 cells/well in 96 well plates in serum free RPMI for 1 hour. Contaminating cells were removed by stringent washing in RPMI, and AMs rested in assay media for 1 hour prior to cytokine release experiments. The purity of macrophages obtained by this process was confirmed as >90% by Wright-Giemsa/May-Grünwald staining.

Cytokine release experiments were performed in IMDM containing 0.5% FCS. Compounds were added for 30 minutes, prior to addition of LPS (100 ng/ml final concentration) and cells incubated for 20 hours. Supernatants were harvested and cytokines measured using optEIA ELISA kits (BD Biosciences, Erembodegen, Belgium) according to manufacturer’s instructions. The compounds had no effect on cell viability at the concentrations used as assessed by cellular morphology and/or Wst-1 viability assays.

### Data analysis

To estimate the potency of LPS, concentration-effect curve data were fitted to the following form of the Hill equation:

(1) $$Ε=\frac{\alpha {\left[A\right]}^{\mathrm{nH}}}{{\left[A\right]}^{\mathrm{nH}}+{\left[A\right]}_{50}^{\mathrm{nH}}}$$

in which α, [A]_50_ and n_H_ are the upper asymptote (maximum effect), location (potency) and slope parameters, respectively. [A]_50_ values were assumed to be log-normally distributed and quoted as p[A]_50_ (−log[A]_50_) values.

Similarly, potencies of Budesonide and BIRB-796 were estimated by fitting inhibitory concentration-effect curve data to a modified version of equation (1):

(2) $$Ε=\frac{\alpha {\left[I\right]}^{\mathrm{nH}}}{{\left[I\right]}^{\mathrm{nH}}+{\left[IC\right]}_{50}^{\mathrm{nH}}}$$

in which α, [IC]_50_ and n_H_ are the upper asymptote (maximum effect), location (potency) and slope parameters, respectively. [IC]_50_ values were assumed to be log-normally distributed and quoted as p[IC]_50_ (−log[IC]_50_) values. In all cases the individual concentration-effect curve data was averaged and this mean data fitted to equations 1 and 2. Maximum effects quoted in the results sections are the measured values rather than the values obtained from the fitting procedures. Percentage inhibition was calculated by the equation: ((LPS in presence of compound – basal) ÷ (LPS in presence of vehicle – basal)) × 100. All curve fitting was done using GraphPad Prism software, using a non-linear regression curve fit with least squares fit. Results are expressed and plotted as mean ± s.e. Where appropriate, a two tailed T-test was performed to assess statistical significance. *p* values of <0.05 were considered significant.

## Abbreviations

DMSO: Dimethyl sulfoxide; TNFα: Tumor necrosis factor-alpha; IL-6: Interleukin-6; BIRB-796: 1-(5-tert-Butyl-2-p-tolyl-2H-pyrazol-3-yl)-3-[4-(2-morpholin-4-yl-ethoxy)-naphthalen-1-yl]-urea; COPD: Chronic Obstructive Pulmonary Disease; PDEIV: Phosphodiesterase IV; AM: Alveolar macrophage; FCS: Fetal calf serum; LPS: Lipopolysaccharide; BAL: Bronchoalveolar lavage.

## Competing interests

The authors are current or recent employees of AstraZeneca Plc.

## Authors’ contributions

MJR conducted the experiments and performed the data analysis. IGD contributed to the data analysis. Both authors contributed to the study design and manuscript preparation. All authors read and approved the final manuscript.

## Pre-publication history

The pre-publication history for this paper can be accessed here:

http://www.biomedcentral.com/2050-6511/13/15/prepub
